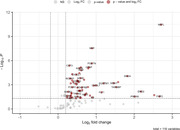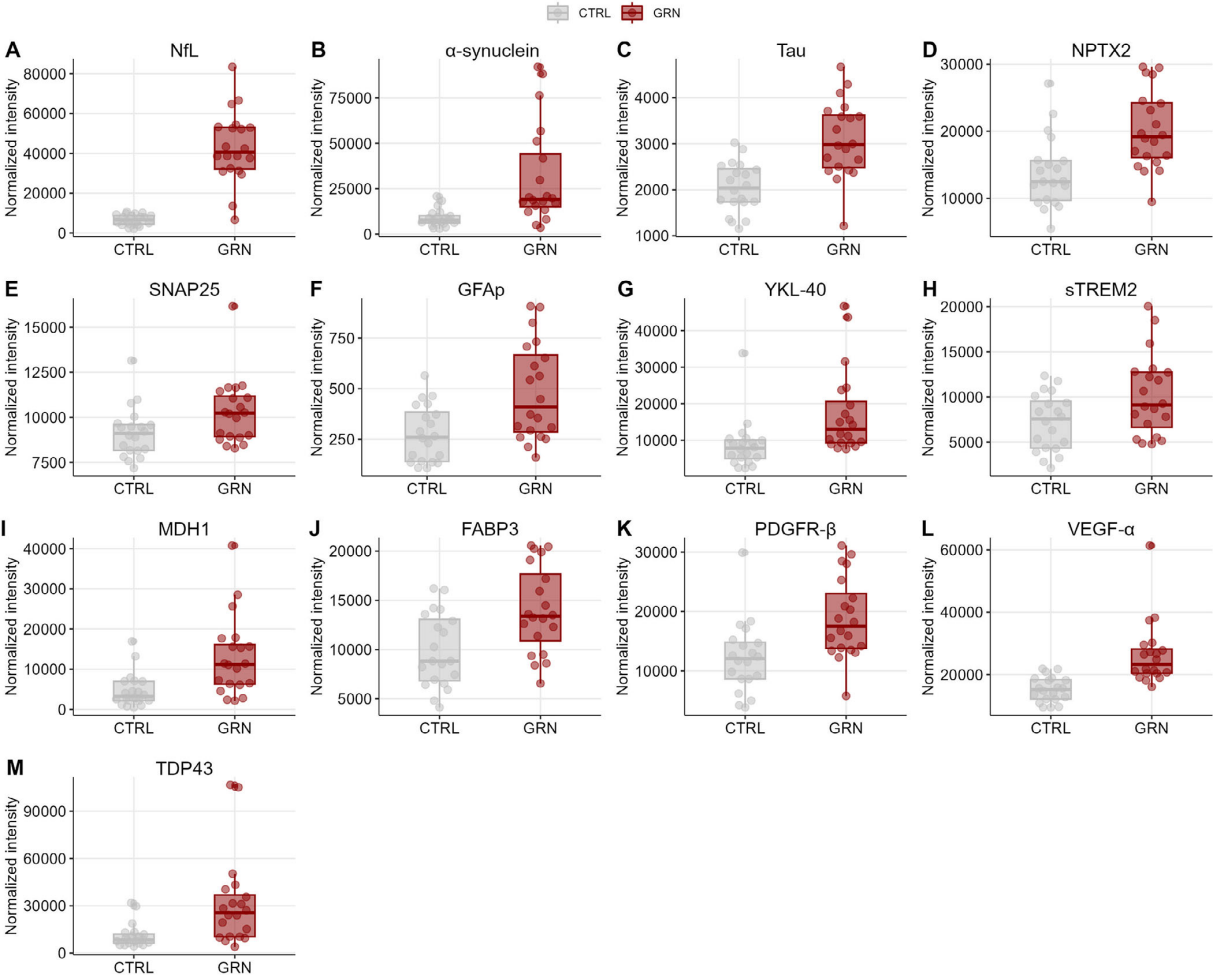# Plasma proteomic changes in carriers of progranulin mutations on the novel NULISA platform

**DOI:** 10.1002/alz.092348

**Published:** 2025-01-09

**Authors:** Joel Simrén, Andrea L. Benedet, Bingqing Zhang, Guglielmo Di Molfetta, Valentina Cantoni, Ilenia Libri, Jasmine Rivolta, Kaj Blennow, Henrik Zetterberg, Barbara Borroni, Nicholas J. Ashton

**Affiliations:** ^1^ Clinical Neurochemistry Laboratory, Sahlgrenska University Hospital, Mölndal Sweden; ^2^ Institute of Neuroscience and Physiology, Department of Psychiatry and Neurochemistry, The Sahlgrenska Academy at University of Gothenburg, Mölndal Sweden; ^3^ Department of Psychiatry and Neurochemistry, Institute of Neuroscience and Physiology, The Sahlgrenska Academy, University of Gothenburg, Mölndal, Gothenburg Sweden; ^4^ Alamar Biosciences, Fremont, CA USA; ^5^ Department of Psychiatry and Neurochemistry, Institute of Neuroscience and Physiology, The Sahlgrenska Academy, University of Gothenburg, Mölndal Sweden; ^6^ University of Brescia, Brescia, Brescia Italy; ^7^ University of Brescia, Brescia Italy; ^8^ Institute of Neuroscience and Physiology, Department of Psychiatry and Neurochemistry, The Sahlgrenska Academy at University of Gothenburg, Mölndal, ‐ Sweden; ^9^ Department of Neurodegenerative Disease, UCL Queen Square Institute of Neurology, University College London, London, ‐ UK; ^10^ Wisconsin Alzheimer's Disease Research Center, University of Wisconsin School of Medicine and Public Health, Madison, WI USA; ^11^ Hong Kong Center for Neurodegenerative Diseases, Clear Water Bay Hong Kong; ^12^ UK Dementia Research Institute at UCL, London UK; ^13^ Centre for Age‐Related Medicine, Stavanger University Hospital, Stavanger Norway; ^14^ King’s College London, Institute of Psychiatry, Psychology & Neuroscience, Maurice Wohl Clinical Neuroscience Institute, London UK

## Abstract

**Background:**

With no effective therapy targeting the pathology of genetic frontotemporal lobar degeneration (FTLD), there is a need for easily accessible biomarkers enabling the development of therapeutic agents and for clinical diagnostics. Thus, we aimed to investigate the proteomic changes in plasma of progranulin (GRN) mutation carriers using a novel ultrasensitive antibody‐based platform.

**Methods:**

We cross‐sectionally evaluated carriers of pathogenic GRN mutations (GRN+) and age‐ and sex‐matched cognitively healthy non‐carriers (GRN‐) from the University of Brescia. Participants underwent venipuncture, with blood being collected in EDTA tubes to obtain plasma. In these samples, we quantified the proteins included in the “CNS disease panel” on the novel nucleic acid‐linked immuno‐sandwich assay (NULISA) platform (Alamar Biosciences). Biomarker data were log_2_‐transformed prior to statistical analysis, including linear models (age and sex as covariates) comparing protein concentrations between GRN+ and GRN‐. P‐values were adjusted for false discovery rate (FDR). Protein log_2_‐fold changes between GRN+ and GRN‐ were calculated using untransformed data.

**Results:**

We included twenty (2 presymptomatic and 18 symptomatic with median [range] Global CDR plus NACC FTLD scores of 1 [0.5‐3]) GRN+ and twenty GRN‐ (median [range] age 64 [45‐85] years, 65% women). Fifty‐four of the 116 proteins quantified had an adjusted P<0.05 (Figure 1). Significantly altered proteins included markers of neuronal/synaptic injury (neurofilament light [NfL], α‐synuclein, tau, NPTX2 and SNAP‐25, log_2_‐fold changes 0.2‐2.7; Figure 2A‐E), glial activation/neuroinflammation (glial fibrillary acidic protein [GFAp], YKL‐40, MDH1, FABP3, and sTREM2, log_2_‐fold changes 0.5‐1.5; Figure 2F‐J) and blood‐brain barrier dysfunction (PDGFR‐β and VEGFA, log_2_‐fold changes 0.6 and 0.8; Figure 2K‐L). Notably, TDP‐43 was elevated in GRN+ (log_2_‐fold change 1.5, P<0.01; Figure 2M). Notable markers that were unaltered included p‐tau217, Aβ42 and NPTX1.

**Conclusion:**

Using a biomarker panel simultaneously quantifying >100 relevant proteins on the NULISA platform, clear plasma proteomic alterations were observed in GRN mutation carriers. This new methodology has the potential of improving clinical diagnostics and trials, as well as providing insights into the pathophysiology of FTLD due to GRN mutations. Further data will increase the sample size, including >20 presymptomatic carriers, C9orf72 mutation carriers, and a wider range of disease phenotypes, providing opportunities for more detailed analyses.